# Tetracycline induces *wsp* operon expression to promote biofilm formation in *Pseudomonas putida*

**DOI:** 10.1128/aem.01071-24

**Published:** 2024-11-26

**Authors:** Kexin Mu, Meina He, Haozhe Chen, Tong Liu, Ying Fan, Yongxin Tao, Haoqi Feng, Qiaoyun Huang, Yujie Xiao, Wenli Chen

**Affiliations:** 1 National Key Laboratory of Agricultural Microbiology, Huazhong Agriculture University 124443 , Wuhan, China; Shanghai Jiao Tong University, Shanghai, China

**Keywords:** tetracycline, biofilm, c-di-GMP, Wsp system, RpoS, transcriptional regulation, *Pseudomonas putida*

## Abstract

The overuse and wanton discharge of antibiotics produces a threat to bacteria in the environment, which, in turn, stimulates the more rapid emergence of antibiotic-resistant bacteria. *Pseudomonas putida* actively forms biofilms to protect the population under tetracycline stress, but the molecular mechanism remains unclear. This study found that tetracycline at sub-minimal inhibitory concentrations increased cyclic diguanylate (c-di-GMP), a second messenger that positively regulates biofilm formation. Four c-di-GMP-metabolizing proteins were found to be involved in the tetracycline-mediated biofilm promotion, including DibA, WspR, PP_3242, and PP_3319. Among them, the diguanylate cyclase WspR displayed the most significant effect on c-di-GMP level and biofilm formation. *WspR* belongs to the *wsp* operon comprising seven genes (*wspA–wspF* and *wspR*). The *wsp* operon contained six promoters, including one major start promoter (P*_wspA_*) and five internal promoters (P*_wspB_*, P*_wspC_*, P*_wspD_*, P*_wspF_*, and P*_wspR_*), and tetracycline promoted the activity of P*_wspA_*. The stress-response sigma factor RpoS directly bound to P*_wspA_* and positively regulated its activity under tetracycline stress. Moreover, RpoS was required for tetracycline to induce P*_wspA_* activity and promote biofilm formation. Our results enrich the transcriptional regulation of the *wsp* operon and reveal the mechanism by which tetracycline promotes biofilm formation in *P. putida*.

**IMPORTANCE:**

The overuse and wanton discharge of antibiotics produces a threat to bacteria in the environment, which, in turn, stimulates the more rapid emergence of antibiotic-resistant bacteria. The *Pseudomonas putida* actively forms biofilm against antibiotic threats, but the mechanism remains unclear. Here, our results showed that tetracycline treatment at sub-minimal inhibitory concentrations could induce the expression of the Wsp system via the sigma factor RpoS in *P. putida*, resulting in elevated c-di-GMP levels, which leads to increased biofilm formation. The *wsp* operon contains one major promoter and five internal promoters, and RpoS directly binds to the major promoter to promote its activity.

## INTRODUCTION

Tetracycline is a broad-spectrum antibiotic, and it is widely used for human therapy, veterinary purposes, and agricultural purposes (1). Due to its overuse and wanton discharge, tetracycline has become a common compound in different ecosystems, and the resulting tetracycline pollution poses a threat to environmental microbial biodiversity (2–4). Biofilms are microbial aggregation membranes formed when microorganisms attach to the surfaces of living or nonliving things (5). Forming biofilm improves the ability of bacteria to cope with antibiotic threats and other adverse conditions (5, 6). The relationship between biofilm formation and antibiotics attracts widespread attention. Both aminoglycoside antibiotics and β-lactam antibiotics at sub-minimal inhibitory concentrations (sub-MICs) are found to promote biofilm formation (7–10). However, the mechanism by which antibiotics promote biofilm formation has not been studied well.

Cyclic diguanylate (c-di-GMP) is a ubiquitous bacterial second messenger that regulates diverse physiological processes (11). The most widely studied process regulated by c-di-GMP is the plankton-to-biofilm lifestyle transition. Generally, high-level c-di-GMP favors biofilm matrix production (such as adhesive proteins and exopolysaccharides), leading to a biofilm lifestyle. In contrast, low-level c-di-GMP promotes flagellar synthesis and motility, resulting in a plankton lifestyle (11). Regulation of biofilm formation by c-di-GMP occurs at both transcriptional and post-transcriptional levels in *Pseudomonas* species. At the transcriptional level, c-di-GMP promotes the transcription of genes/operons involved in biofilm matrix synthesis via the receptor FleQ (12–14). At the post-transcriptional level, cleavage of the key biofilm matrix component (adhesion LapA) is controlled by a c-di-GMP-mediated system mainly consisting of a c-di-GMP receptor (LapD) and a protein protease (LapG) (15–17).

In *Pseudomonas* species, the Wsp (wrinkly spreader phenotype) signal transduction system encoded by the *wsp* operon is a conserved and vital c-di-GMP-producing system that consists of seven proteins, including WspA, WspB, WspC, WspD, WspE, WspF, and WspR (18). Among these proteins, WspR is a diguanylate cyclase (DGC) with c-di-GMP-synthesizing ability, and it functions as a primary output component of the Wsp system (19). The cytomembrane located methyl-accepting chemotaxis protein WspA senses and transfers surface/external signals to the kinases WspE through the protein complex WspB/WspD. WspE then autophosphorylates and transfers the phosphorylation signal to WspR (20). The phosphorylation enhances the DGC activity of WspR and causes elevated c-di-GMP levels (18, 21). WspF (a homolog protein of methylesterase CheB) inhibits the transphosphorylation of WspR via modulating the methylation state of WspA (22). *WspR* deletion abolishes the function of the Wsp system. In contrast, *wspF* deletion locks the Wsp system in a constantly activated state in which WspR is continuously phosphorylated to produce c-di-GMP (18). In addition, a spontaneous in-frame deletion in WspA (WspA_Δ280-307_) also locks the Wsp signal transduction system into a constitutively active state (23).

Previous studies have revealed that surface growth activates the Wsp system, resulting in elevated c-di-GMP levels and biofilm formation (18, 22). Recent evidence shows that the Wsp system is activated by misfolded proteins in periplasm caused by surface-induced cell envelope stress (24). Except for biofilm formation, the Wsp system has also been reported to regulate other processes, such as c-di-GMP heterogeneity during surface sensing and the expression of the Type VI secretion system (25, 26). Besides, in *Lysobacter enzymogenes* (a non-flagellated, predatory soil bacterium), a homologous Wsp system is reprogramed to inhibit the biosynthesis of heat-stable antifungal factor (HSAF) via c-di-GMP signaling (27). These results show that the Wsp system is a crucial c-di-GMP-producing system involved in biofilm formation and several other cellular processes. However, there is little research on the transcriptional regulation of the *wsp* operon under certain conditions/stimuli and the regulatory elements involved in the transcription of the *wsp* operon.

*Pseudomonas putida* is a Gram-negative bacterium with complicated metabolic networks and has been widely applied in diverse biotechnological fields (28–31). *P. putida* forms biofilm to adapt to complex environmental conditions. Bacteria inside biofilm are much more resistant to harmful conditions than planktonic forms (32). A previous study showed that *P. putida* formed biofilms in response to antibiotic stress (tetracycline and several other antibiotics) (10). However, the molecular mechanism(s) behind the antibiotic-mediated biofilm enhancement remains unclear. This study discovered that tetracycline increased the c-di-GMP level in *P. putida*, and WspR was involved in the tetracycline-mediated biofilm promotion. Moreover, we found that the stress-response sigma factor RpoS directly bound to the major promoter of the *wsp* operon and positively regulated its activity under tetracycline stress, leading to increased biofilm formation.

## RESULTS

### Tetracycline promotes biofilm formation and c-di-GMP content in *P. putida*

**Fig 1:**
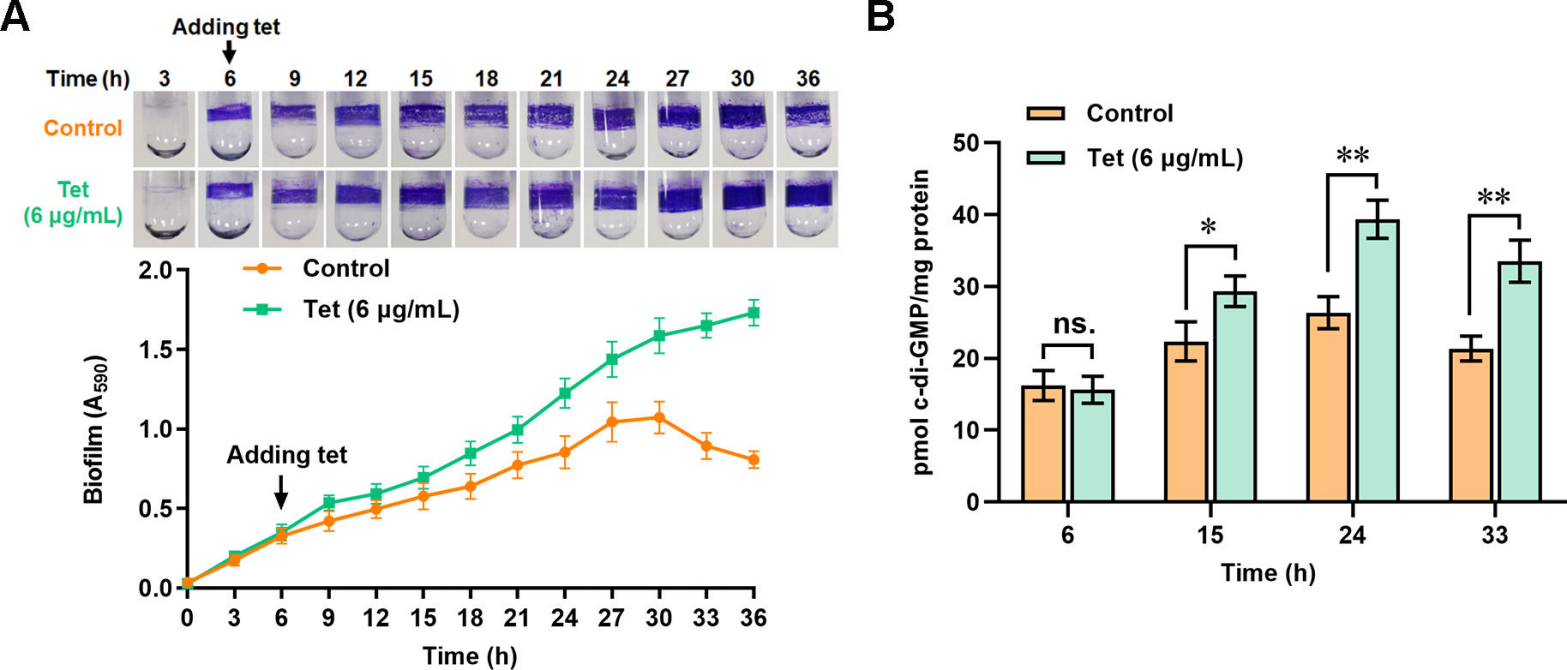
Tetracycline treatment increases biofilm formation and c-di-GMP level. (**A**) Influence of tetracycline treatment on biofilm formation of *P. putida* on glass tubes. The biofilm on the tubes was stained with crystal violet, and the time above the tubes indicated the incubation time (hour). Biofilm biomass in the presence (green line) and absence (orange line) of tetracycline during 36 h of incubation was shown below. The strain was cultured for 6 h before adding tetracycline (final concentration 6 µg/mL). (**B**) Influence of tetracycline treatment on c-di-GMP level at indicated incubation time. The c-di-GMP was extracted and measured using LC-MS/MS, as described in the method section. The results are the average of three independent assays. The data represent mean values with standard deviations (*P < 0.05, **P < 0.01). “ns.” represents no statistical significance between the two compared data. Student’s *t*-test was used to compare c-di-GMP levels in two strains.

Previous studies showed that antibiotics (such as tetracycline) at sub-MICs stimulated biofilm formation in *P. putida* (10). Since c-di-GMP played a vital role in regulating biofilm formation (11), we speculated that tetracycline might modulate biofilm formation by changing the c-di-GMP level. To test this hypothesis, we investigated the influence of tetracycline on biofilm formation and c-di-GMP level. The strain was cultured for 6 h before adding tetracycline (final concentration 6 µg/mL). Similar to previous results, the biofilm formation was more robust in the tetracycline-treated group than in the control group (Fig. 1A). Besides, the biofilm of the control group began to collapse, but the biofilm of the tetracycline-treated group remained intact and strong after 30 h incubation (Fig. 1A). These results indicated that tetracycline promoted biofilm formation and prevented biofilm dispersal. In terms of c-di-GMP detection, the c-di-GMP of the control group and the tetracycline-treated group was extracted at 6, 15, 24, and 33 h of culture and then measured by using liquid chromatography-tandem mass spectrometry (LC-MS/MS). The results showed that the c-di-GMP level in the tetracycline-treated group was higher than that of the control group at 15, 24, and 33 h of culture, while no noticeable difference was observed between the two groups at the time of tetracycline addition (6 h) (Fig. 1B), indicating that the addition of tetracycline increased c-di-GMP level in *P. putida*.

### Four c-di-GMP-metabolizing proteins are involved in the tetracycline-mediated biofilm formation

**Fig 2:**
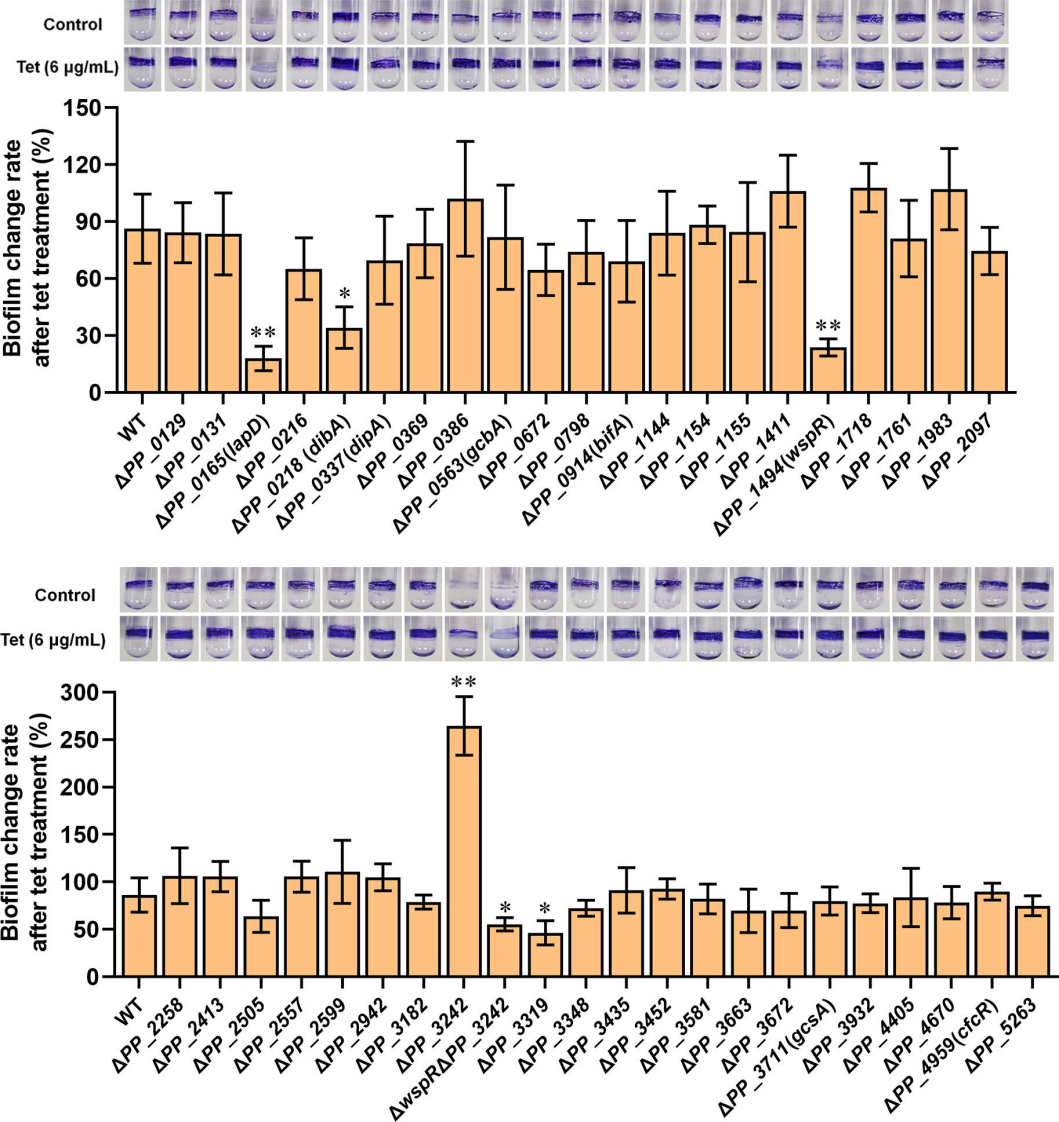
The effects of c-di-GMP metabolism-related genes on the tetracycline-mediated biofilm promotion. Photos of biofilm on glass tubes in The absence and presence of tetracycline were shown above. The biofilm on glass tubes was assayed 10 h after adding 6 µg/mL tetracycline or an equal amount of control solvent (ethanol). The biofilm change rate of indicated strains after tetracycline treatment (below) was calculated based on crystal violet stain assay results. The results are the average of three independent assays. The data represent mean values with standard deviations. The asterisk above the column represents a significant difference between the indicated mutant and wild-type strain analyzed by Student’s *t*-test (*P < 0.05, **P < 0.01).

The genome of *P. putida* KT2440 contained 42 potential c-di-GMP metabolism-related genes (33). To explore the c-di-GMP-metabolizing genes involved in tetracycline-mediated biofilm promotion, we tested the influence of tetracycline on biofilm formation in 42 mutants, each missing one potential c-di-GMP metabolism-related gene. The results showed that five mutants displayed significantly different biofilm change rates after tetracycline treatment compared with the wild-type strain (WT), including Δ*PP_0165* (Δ*lapD*), Δ*PP_0218* (Δ*dibA*), Δ*PP_1494* (Δ*wspR*), Δ*PP_3242*, and Δ*PP_3319* (Fig. 2). Among the five mutants, Δ*PP_3242* showed an increased biofilm change rate, while the other four mutants showed a decreased biofilm change rate after tetracycline treatment (Fig. 2), suggesting that *PP_3242* played a negative role, while *lapD*, *dibA*, *wspR*, and *PP_3319* played a positive role in the tetracycline-induced biofilm promotion. Moreover, of the four genes that played a positive role, *lapD* and *wspR* played a more critical role in biofilm formation than *dibA* and *PP_3319* (Fig. 2). LapD was a degenerated c-di-GMP-metabolizing protein without enzyme activity, and it functioned as a c-di-GMP receptor to control biofilm formation (17). WspR was a DGC with c-di-GMP-synthesizing ability, and it played a vital role in c-di-GMP level maintenance and biofilm formation in *Pseudomonas* species (19, 24, 33). To further interpret the role of *wspR* in the tetracycline-mediated biofilm promotion, we removed the negative influence gene (*PP_3242*) by constructing a *wspR PP_3242* double deletion mutant (Δ*wspR*Δ*PP_3242*) and tested its biofilm change rate after tetracycline treatment. The result exhibited that tetracycline treatment promoted biofilm formation of Δ*wspR*Δ*PP_3242* with a biofilm change rate close to 55%, which was lower than that in Δ*PP_3242* (about 260%) but higher than that in Δ*wspR* (about 23%) (Fig. 2). These results revealed a positive role of *wspR* in the tetracycline-enhanced biofilm formation. Therefore, our subsequent research focused on the role of WspR in tetracycline-mediated biofilm promotion.

### Tetracycline treatment increases *wspR* expression

**Fig 3:**
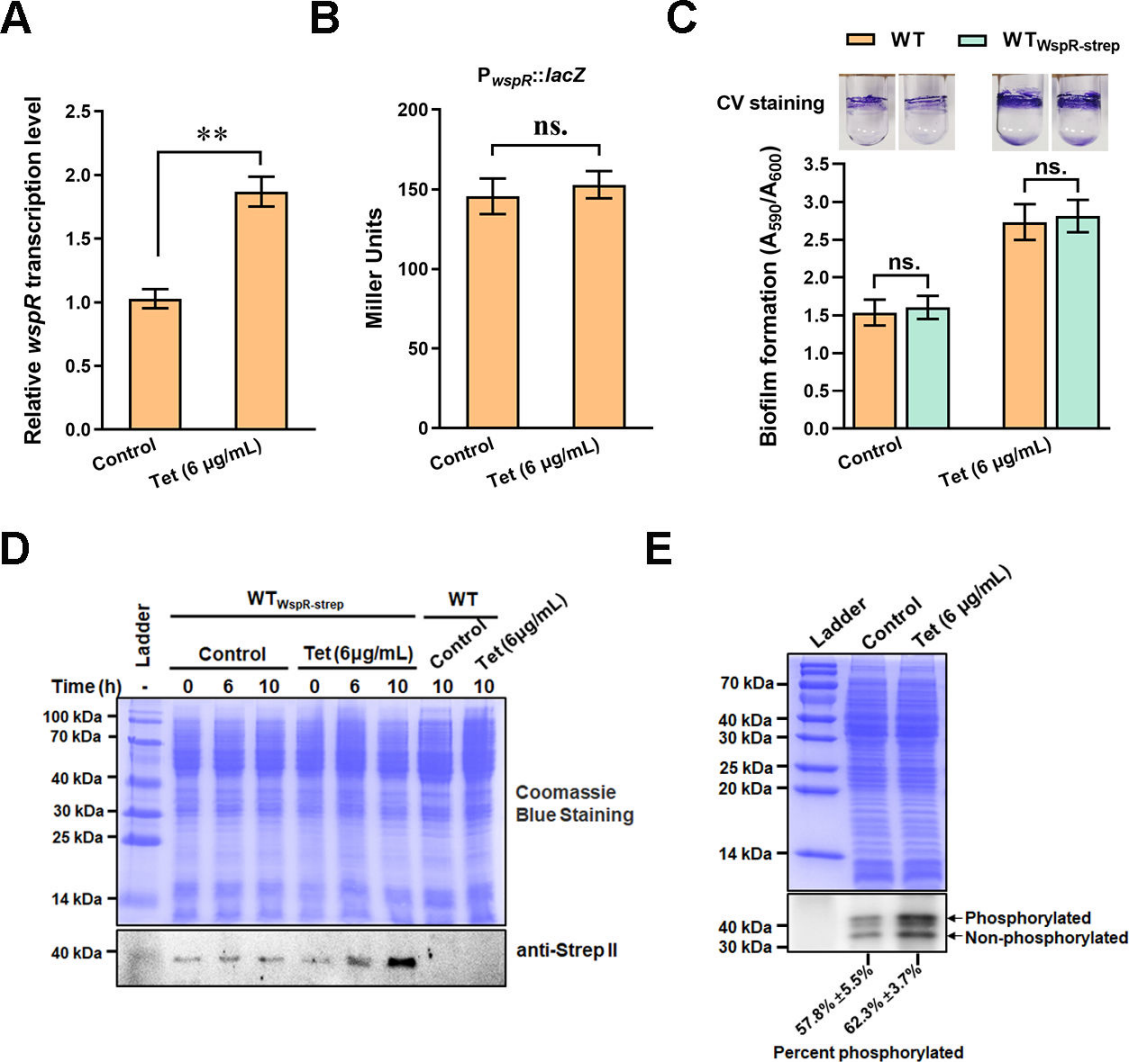
Tetracycline treatment promotes *wspR* expression. (**A**) The relative expression level of *wspR* in the presence and absence of tetracycline was tested by qPCR. (**B**) Promoter-*lacZ* fusion reporter tested the activity of P*_wspR_* in the presence and absence of tetracycline. (**C**) Biofilm formation of WT and WT containing Strep II tag WspR (WT_WspR-strep_) in the presence and absence of tetracycline. The biofilm on glass tubes was assayed 10 hours after adding 6 µg/mL tetracycline or an equal amount of control solvent (ethanol). The results in panels A, B, and C are the average of three independent assays. The data represent mean values with standard deviations (**P < 0.01). “ns.” represents no statistically significant between the two compared data. Student’s *t*-test was used to compare two data groups. (**D**) Detection of WspR in the presence and absence of tetracycline using Western blot. Time represents the incubation time after the addition of tetracycline or control solvent. (**E**) Phos-tag SDS-PAGE and Western blot tested the phosphorylation rate of WspR in the presence and absence of tetracycline. WspR in its phosphorylated state and unphosphorylated state are indicated in the gel. The percentage of phosphorylated WspR to total WspR in each lane was calculated using Image J software, shown below the lane. Total protein from indicated strains in panels D and E were resolved by SDS-PAGE (upper panel). Immunoblotting in panels D and E was performed with an antibody against the Strep II tag (bottom panel).

To investigate how WspR was involved in the tetracycline-mediated biofilm promotion, we made two hypothesizes: (i) tetracycline-treatment induces the expression of *wspR*, leading to increased c-di-GMP level and biofilm formation; (ii) tetracycline-treatment activates WspR via increasing the phosphorylation of WspR, leading to increased c-di-GMP level and biofilm formation. To test the first hypothesis, we investigated the influence of tetracycline on *wspR* transcription using quantitative PCR (qPCR). The results showed that the mRNA level of *wspR* in tetracycline-treated strains was about twice that of the control group, indicating that tetracycline treatment increased the transcription of *wspR* (Fig. 3A). We also used promoter-*lacZ* fusion reporter to investigate the effect of tetracycline on *wspR* expression. The upstream fragment (about 500 bp) of *wspR* containing the *wspR* promoter (P*_wspR_*) was amplified and fused to a *lacZ* reporter. However, results from the LacZ activity assay revealed no apparent difference in LacZ activity between the tetracycline-treated and the control groups (Fig. 3B), suggesting that tetracycline treatment had no apparent influence on the activity of P*_wspR_*.

To further test the effect of tetracycline on *wspR* expression, we performed a western-blot assay to compare the amount of WspR in the presence and absence of tetracycline. We fused a Strep II tag-encoding sequence into the carboxyl-terminal of WspR on the genome. The obtained strain (termed as WT_WspR-strep_) showed a similar biofilm as the wild-type strain without Strep II tag both in the presence and absence of tetracycline (Fig. 3C), indicating that the Strep II tag on WspR had no apparent effect on the physiological activity of WspR. We then used Western blot assay to detect Strep II-tagged WspR in the control and tetracycline-treated groups. As shown in Fig. 3D, WspR was detected in the total protein of WT_WspR-strep II_ but was undetectable in WT without Strep II tag in a similar amount of total protein. Moreover, the amount of WspR in WT_WspR-strep II_ from the tetracycline treatment group was higher than that from the control group (Fig. 3D), indicating that tetracycline treatment increased the amount of WspR. To test the second hypothesis, we used Phos-tag gel to assess the effect of tetracycline treatment on the phosphorylation of WspR. The results revealed no apparent WspR phosphorylation ratio difference between the tetracycline-treated and the control groups (Fig. 3E). Together, these results indicated that tetracycline treatment increased *wspR* expression, leading to increased WspR protein levels in *P. putida*.

### Tetracycline promotes the activity of the major promoter in *wsp* operon

**Fig 4:**
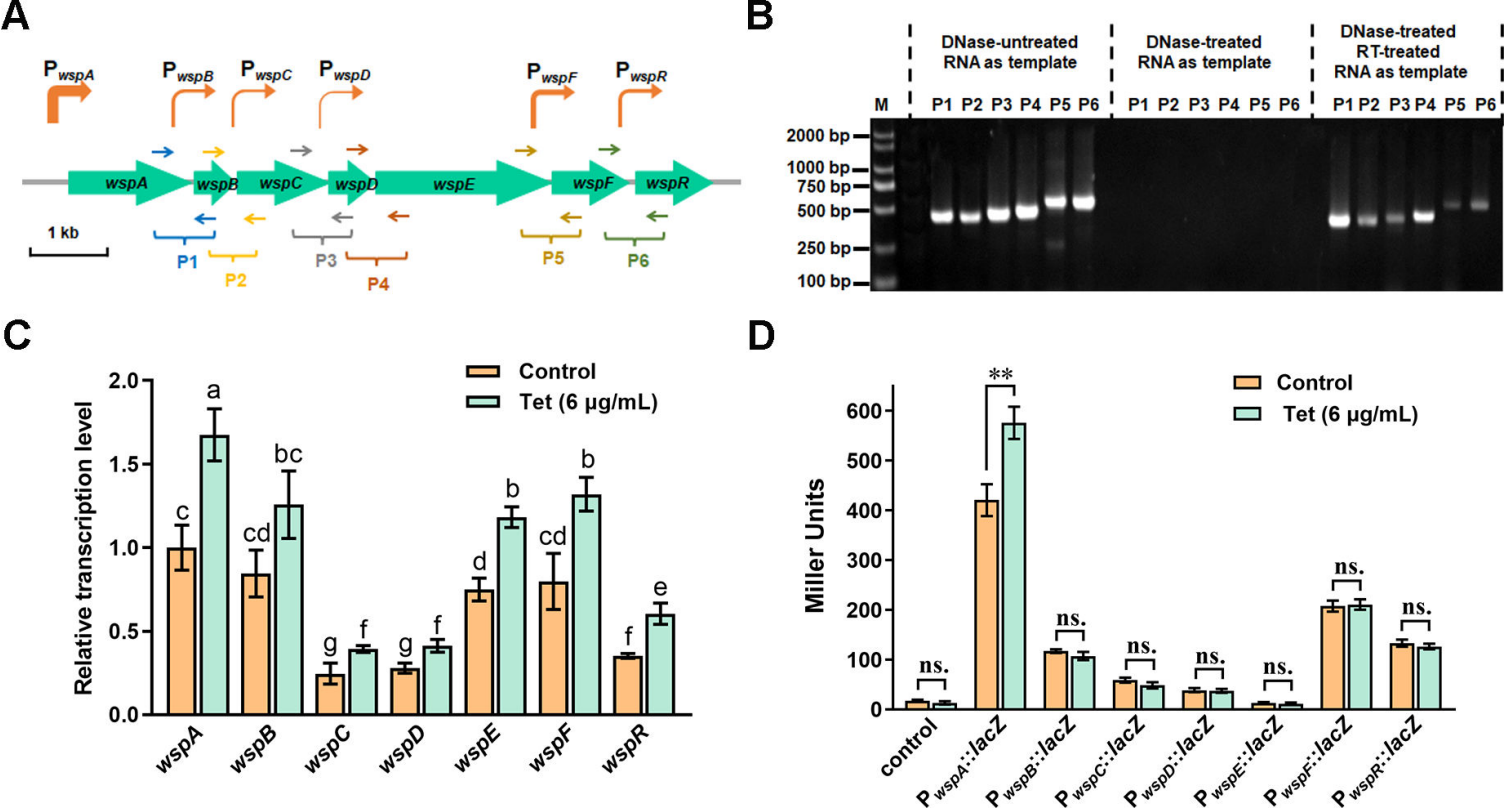
The effect of tetracycline treatment on the activity of *wsp* promoters. (**A**) Schematic drawing of the gene and promoter distribution in the *wsp* operon of *P. putida* KT2440. The green arrows represent genes. The orange arrows represent promoters, and the arrow’s thickness corresponds to the strength of promoter activity. Short arrows indicate primer pairs (**P1–P6**) used for the co-transcription test. A scale bar of 1 kilobase (kb) is indicated. (**B**) RT-PCR tests co-transcription of the seven genes in the *wsp* operon. The type of templates used in PCR is shown above the gel. RT-treated represents reverse transcriptase-treated. M represents the DNA ladder. P1–P6 represent PCR product amplified with associated primer pairs as indicated in panel A. (**C**) Relative transcriptional levels of the seven *wsp* genes in the presence and absence of tetracycline. The transcriptional level of *wspA* is set as 1, and the transcriptional levels of the other six genes are shown as the relative transcriptional level of *wspA*. ANOVA was used to compare the transcription level of different genes. (**D**) The activity of *wsp* promoters in the presence and absence of tetracycline. The promoter activity is indicated by LacZ activity. The vector containing promoter-less *lacZ* is used as the negative control. Student’s *t*-test was used to compare LacZ activity from two strains. In panels C and D, error bars represent standard deviations, and significant differences (*P* < 0.05) are denoted by the lowercase letters above each bar (panel C) or asterisk above the column (panel D) (**P < 0.01). “ns.” represents no statistically significant between two compared data.

According to the gene annotation in the Pseudomonas Genome Database (34), the *wsp* operon of *P. putida* KT2440 contains seven adjacent genes transcribed in the same direction, including *wspA* (*PP_1488*), *wspB* (*PP_1489*), *wspC* (*PP_1490*), *wspD* (*PP_1491*), *wspE* (*PP_1492*), *wspF* (*PP_1493*), and *wspR* (*PP_1494*) (Fig. 4A). The seven genes are shortly overlapped or separated with a short distance (5–48 bp). We extracted total RNA from wild-type KT2440 and performed reverse transcription PCR (RT-PCR) to determine whether the seven genes were co-transcribed. Six primer pairs between adjacent genes were designed and used for the RT-PCR. The result revealed that the seven genes in the *wsp* operon were co-transcribed (Fig. 4B). Then, we performed absolute quantitative PCR to test and compare the transcriptional levels of the seven *wsp* genes in the presence and absence of tetracycline. The result showed that tetracycline treatment promoted the expression of all seven genes (Fig. 4C). Moreover, the transcriptional levels of the seven genes varied significantly under both conditions and were arranged as follows: *wspA* > *wspB*/*wspF* > *wspE* > wspR > *wspC*/*wspD* (Fig. 4C). The transcriptional level of *wspA* was about fourfold higher than that of *wspC*/*wspD* in the absence of tetracycline (Fig. 4C). The differential transcriptional levels indicated that internal promoters existed in *wsp* operon.

To test if internal promoters existed in the *wsp* operon, we amplified the upstream regions of the seven genes and then tested their promoter activities with a promoter-less *lacZ* reporter. The LacZ activity assay characterized the promoter activity of each upstream region. The empty plasmid was used as a negative control. The result showed that except for the upstream region of *wspE*, all the other six upstream regions showed apparent promoter activity (Fig. 4D). The upstream region of *wspA* (the major start promoter, termed as P*_wspA_*) displayed the strongest promoter activity, while the upstream region of *wspD* (termed as P*_wspD_*) showed the weakest promoter activity (Fig. 4D). Based on the LacZ activity, the promoter activities of the six upstream regions were arranged as follows: P*_wspA_* > P*_wspF_* > P*_wspR_* > P*_wspB_* > P*_wspC_* > P*_wspD_* (Fig. 4D).

To figure out how tetracycline promotes *wspR* expression, we tested the influence of tetracycline on the promoter activity of the six *wsp* promoters. The strain was cultured for 6 h before adding tetracycline, and LacZ activity was measured at 10 h of culture after tetracycline addition. The results demonstrated that the addition of tetracycline increased the promoter activity of P*_wspA_* but had no evident influence on the promoter activity of the other five promoters (Fig. 4D). Together, these results indicated that tetracycline promoted the activity of the major *wsp* promoter P*_wspA_*.

### RpoS directly and positively regulates the activity of P*_wspA_*

**Fig 5:**
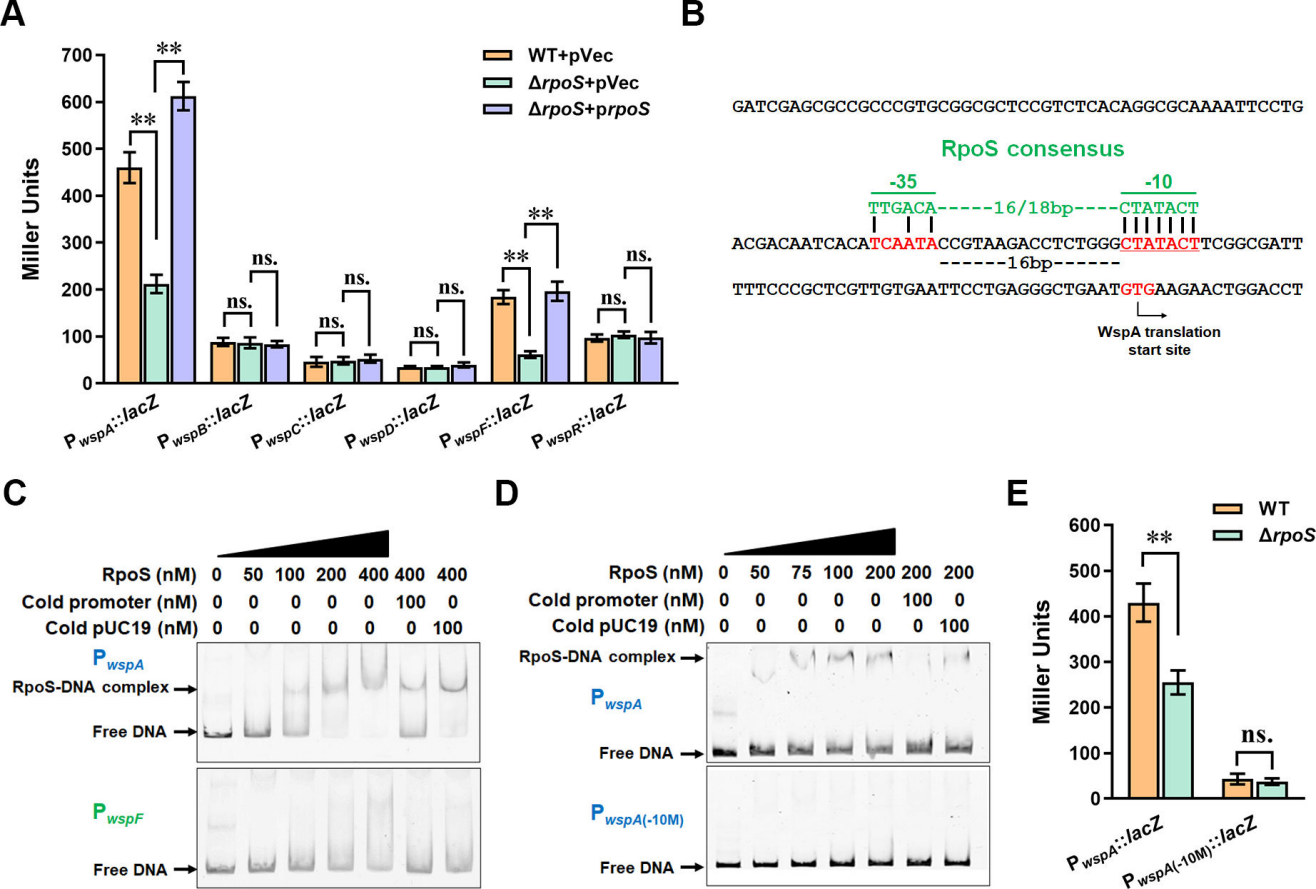
RpoS directly and positively regulates the activity of P*_wspA_*. (**A**) The effect of RpoS on activities of the six *wsp* promoters. WT + pVec represents the wild-type strain containing the control vector, Δ*rpoS* + pVec represents the *rpoS* deletion mutant containing the control vector, Δ*rpoS* + p*rpoS* represents the complementation strain of Δ*rpoS*. (**B**) The nucleotide sequence of the *wspA* promoter region. The GTG initiation codon of WspA is red and indicated with an arrow. The RpoS consensus motif is green, and the corresponding region in the *wspA* promoter is shown in red. (**C**) Binding of RpoS to P*_wspA_* and P*_wspF_*. The concentration of RpoS and unlabeled specific/non-specific DNA (cold promoter/pUC19) is indicated above each lane. (**D**) Binding of RpoS to the mutated *wspA* promoter (P*_wspA_*_(-10M)_). P*_wspA_* represents the native *wspA* promoter. P*_wspA_*_(-10M)_ represents the P*_wspA_* promoter containing mutations in the RpoS motif sequence (from CTATACT to TCGGCAC). The migration of free DNA and RpoS-DNA complex in panels C and D was indicated by arrows. (**E**) The activities of wild-type *wspA* promoter (P*_wspA_::lacZ*) and mutated *wspA* promoter (P*_wspA_*_(-10M)_::*lacZ*) in WT and Δ*rpoS*. The results in panels A and E are the average of three independent assays. The data represent mean values with standard deviations (**P < 0.01). “ns.” represents none statistically significant between the two compared strains. Student’s *t*-test was used to compare LacZ activity from two strains.

Our previous study revealed that the deletion of the sigma factor RpoS decreased *wspR* expression in *P. putida* (33). The sigma factor RpoS is a stress response regulator critical to bacterial survival under stress conditions (35, 36). We speculated that tetracycline treatment promoted Wsp system expression via RpoS. Thus, we tested the effect of RpoS on the activities of the six *wsp* promoters. The results exhibited that deletion of *rpoS* (Δ*rpoS* + pVec) led to a decrease in the activities of P*_wspA_* and P*_wspF_* while showing no evident impact on the activities of the other four promoters (Fig. 5A). Complementation with multicopy plasmid containing *rpoS* (Δ*rpoS* + p*rpoS*) restored the activities of P*_wspA_* and P*_wspF_* (Fig. 5A). RpoS recognizes and binds to a conserved motif “CTATACT” in the −10 region of the target promoter (37). We analyzed the sequences of P*_wspA_* and P*_wspF_* and found a conserved RpoS motif in P*_wspA_* but not in P*_wspF_* (Fig. 5B). To explore whether RpoS regulated P*_wspA_* via binding to the conserved motif, we performed electrophoretic mobility shift assay (EMSA) to test the interaction between RpoS and P*_wspA_*. The presence of RpoS (final concentration: 100 nM or larger) caused a shift of P*_wspA_* on the gel (Fig. 5C, lanes 3–5). The shift of P*_wspA_* was abolished by the addition of 10-fold unlabeled specific DNA (cold promoter) (Fig. 5C, lane 6), but not 10-fold unlabeled non-specific DNA (cold pUC19 fragment) (Fig. 5C, lane 7), indicating that RpoS specifically bound to P*_wspA_*. Mutation of the conserved motif in P*_wspA_* (from CTATACT to TCGGCAC) abolished the binding of RpoS to P*_wspA_* (Fig. 5D). Meanwhile, mutation of the conserved motif significantly decreased P*_wspA_* activity and abolished the effect of *rpoS* deletion on P*_wspA_* activity (Fig. 5E), suggesting that the conserved RpoS motif played a vital role in P*_wspA_* activity. We also tested the interaction between RpoS and P*_wspF_* using EMSA. The results showed that RpoS showed weak and unspecific binding to P*_wspF_* (Fig. 5C), indicating that RpoS indirectly regulated the activity of P*_wspF_*. These results demonstrated that RpoS directly and positively regulated the expression of the *wsp* operon by binding to the conserved RpoS motif in P*_wspA_*.

### RpoS is required for the tetracycline-mediated biofilm formation

**Fig 6:**
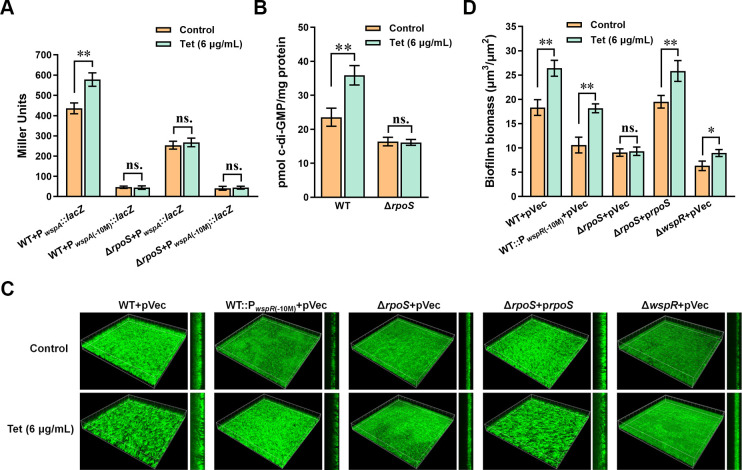
RpoS is required for the tetracycline-mediated biofilm increase. (**A**) P*_wspA_*/P*_wspA_*_(-10M)_ activity in WT and Δ*rpoS* in the absence and presence of tetracycline. (**B**) The impact of tetracycline on c-di-GMP level in WT and Δ*rpoS*. The c-di-GMP was extracted and measured using LC-MS/MS. (**C**) Representative confocal images of biofilm growth under flowing conditions in the absence and presence of tetracycline, as described in Materials and Methods. The thickness of the biofilm is shown on the right. All tested strains contained pBBR403-*egfp*. (**D**) Biofilm biomasses of strains in CLSM image stacks quantified by COMSTAT2. The results of panels A, B, and D are the average of three independent assays. The data represent mean values with standard deviations (*P < 0.05, **P < 0.01). “ns.” represents no statistical significance between the two compared data analyzed by student’s *t*-test.

To investigate the role of RpoS in the tetracycline-mediated P*_wspA_* promotion, we compared the difference in the activity of P*_wspA_*/P*_wspA_*_(-10M)_ in the presence and absence of tetracycline in WT and Δ*rpoS*. The results showed that tetracycline treatment promoted P*_wspA_* activity in WT but failed to affect P*_wspA_* activity in Δ*rpoS* (Fig. 6A). Mutation of the RpoS-binding motif in P*_wspA_* decreased the promoter activity and eliminated the regulation of RpoS on the promoter. These results implied that RpoS was required for the tetracycline-mediated P*_wspA_* promotion. Results from c-di-GMP level measurement demonstrated that Δ*rpoS* contained a lower c-di-GMP level than WT, and the effect of tetracycline on c-di-GMP level was abolished in Δ*rpoS* (Fig. 6B). We further used confocal laser scanning microscopy (CLSM) to investigate the role of RpoS and WspR in the tetracycline-mediated biofilm formation in a flow chamber system. The results showed that tetracycline treatment increased the biofilm formation in WT, with the average biomass of tetracycline-treated WT being about 1.5-fold that of the control WT (Fig. 6C and D). In comparison, Δ*rpoS* formed a thinner biofilm than WT, but the biofilm of Δ*rpoS* showed no apparent difference between the tetracycline-treated group and the control group (Fig. 6C and D), suggesting that the effect of tetracycline on biofilm formation was abolished in Δ*rpoS*. The enhancement effect of tetracycline on biofilm formation was restored by complementation of Δ*rpoS* with a multicopy plasmid containing *rpoS* (Fig. 6C and D). The mutation of the RpoS-binding motif in P*_wspA_* (WT::P*_wspR_*_(-10M)_) decreased biofilm formation, but the promotion effect of tetracycline on biofilm was still significant, only to a lesser extent than that in WT (Fig. 6C and D). Besides, the biofilm formation of Δ*wspR* was the lowest among the tested strains, with the average biomass of Δ*wspR* being approximately 1/3 of that of WT, and tetracycline treatment increased the biofilm formation in Δ*wspR*, but to a lesser extent than in WT (Fig. 6C and D). These results indicated that RpoS and WspR positively regulated biofilm formation, and the increased biofilm formation under tetracycline treatment was RpoS-dependent in *P. putida*.

## DISCUSSION

Biofilms are assemblages of surface-attached microbial cells embedded in a self-produced extracellular matrix (32). Bacteria inside biofilm are much more resistant to antimicrobial agents than planktonic forms (32, 38). A previous study showed that tetracycline and several other antibiotics strongly stimulated biofilm formation in *P. putida* (10), but the mechanism remains unclear. Our result revealed a relationship between the tetracycline-mediated biofilm formation and the second messenger c-di-GMP in *P. putida*. Tetracycline treatment caused increased biofilm formation, accompanied by increased c-di-GMP levels (Fig. 1A and B). *P. putida* contains a complex c-di-GMP metabolic network involving 42 potential c-di-GMP metabolizing proteins (33). Among the 42 proteins, five proteins were found to be involved in the tetracycline-mediated biofilm formation, including LapD, DibA, WspR, PP_3242, and PP_3319 (Fig. 2). Of these five proteins, except for the non-enzymatic c-di-GMP receptor LapD, WspR played a key and positive role in the tetracycline-mediated biofilm formation. Further analysis revealed that tetracycline treatment promoted the transcription of WspR, leading to an increased WspR protein level, but it showed no apparent influence on the phosphorylation of WspR (Fig. 3).

The Wsp signal transduction system is a conserved and dominant c-di-GMP-producing system involved in biofilm formation and several other cellular processes in *Pseudomonas* species (22, 24, 27, 39). Previous studies concerning the Wsp system mainly focused on its function and activity regulation at the protein level, while the transcriptional regulation of the Wsp system was poorly studied. We found that although the seven genes in the *wsp* operon were co-transcribed (Fig. 4B), their transcription levels were quite different (Fig. 4C). By analyzing the promoter activity of the gene upstream regions, we first revealed that the *P. putida wsp* operon contained six promoters, including one major start promoter and five internal promoters. The existence of internal promoters could explain the discrepancy in gene transcription level in the *wsp* operon. Moreover, tetracycline treatment promoted the activity of P*_wspA_* and had no noticeable effect on the activity of internal promoters (Fig. 4D), but the expression of WspR was increased with tetracycline treatment (Fig. 3A and D). These results suggested that the major operon promoter P*_wspA_* was tetracycline responsive, consistent with the result that RpoS bound to P*_wspA_* to regulate its activity (Fig. 5C and D). Besides, internal promoters increase the operon complexity and strain adaptability by affecting the transcription of the following and neighboring genes and regulating the expression of related genes in response to specific signals (40, 41). Thus, future research on the activity and regulation of these *wsp* promoters would enrich our understanding of the relationship between c-di-GMP signaling and bacterial adaptation.

Our previous results demonstrated that deletion of the stress-responsive sigma factor RpoS caused a decrease in *wspR* transcription (33). Based on previous results, we speculated that tetracycline treatment stimulated biofilm formation by inducing *wspR* expression via RpoS. The result that tetracycline treatment promoted *wspR* expression and caused elevated c-di-GMP levels in an RpoS-dependent manner supported our hypothesis (Fig. 6A and B). Moreover, a RpoS-binding motif was identified in P*_wspA_* (Fig. 5B), and further protein-DNA-binding assay confirmed the direct interaction between RpoS and P*_wspA_* at the RpoS-binding motif (Fig. 5C and D). Besides, the effect of tetracycline on biofilm formation still existed in the *wspR* mutant, but the magnitude of the effect was lower than in WT (Fig. 6C), indicating that there were other factors besides WspR involved in the tetracycline-induced biofilm formation. Consistent with this, RpoS also positively regulated the expression of two biofilm matrix-encoding operons, *pea-*encoding exopolysaccharide Pea, and *lapF-*encoding adhesion LapF (42, 43).

In conclusion, our results showed that tetracycline treatment induced the expression of the Wsp system via RpoS in *P. putida*, resulting in elevated c-di-GMP levels, which led to increased biofilm formation. The *wsp* operon contained one major promoter and five internal promoters, and RpoS directly bound to the major promoter to promote its activity. Our results enriched the transcriptional regulation of the *wsp* operon and revealed the mechanism by which tetracycline promoted biofilm formation in *P. putida*.

## MATERIALS AND METHODS

### Bacterial strains and growth conditions

The strains and plasmids used in this study are listed in Table 1. Unless specifically indicated, *P. putida* and *E. coli* strains used in this study were cultured with Lysogeny Broth (LB) medium at 28°C and 37°C, respectively. Antibiotics were used, when required for plasmid maintenance or transformants screening, at the following concentrations: kanamycin (50 µg/mL), chloramphenicol (25 µg/mL), and gentamycin (40 µg/mL).

**TABLE 1:** Strains and plasmids used in this work

Strain or plasmid	Relevant genotype and/or description	Source or reference
*E. coli* strains		
DH5a	λ-Φ80dlacZΔM15Δ(lacZYA-argF)U196 recA1 endA1 hsdR17(rK- mK-) supE44 thi-1 gyrA relA1	Invitrogen Corp
S17-1/λpir	RK2 tra regulon, pir, host for pir-dependent plasmids	Invitrogen Corp
BL21(DE3)	F- ompT hsdS (rBB-mB-) gal dcm (DE3）	Invitrogen Corp
*P. putida* strains		
WT KT2440	Wild-type KT2440	Lab stock
Δ*rpoS*	Unmarked *rpoS* deletion mutant	(43)
Δ*wspR*	*wspR* deletion mutant, *wspR*::*Km^r^*	(33)
WT+pVec	Wild-type KT2440 harboring empty vector pBAK	This work
WT+p*rpoS*	Wild-type KT2440 harboring empty vector pBAK-*rpoS*	This work
Δ*rpoS*+p*csoR*	*rpoS* deletion mutant containing complementary plasmid pBAK-*rpoS*	This work
Plasmids		
pBBR–*lacZ*	Derived from pBBR1MCS5, harbors a promoterless *lacZ* gene, Gm^r^	Lab stock
P*_wspA_*::*lacZ*	Reporter plasmid with *wspA* promoter ligated to pBBR–*lacZ*	This work
P*_wspA_*_(-10M)_::*lacZ*	Reporter plasmid with mutated *wspA* promoter ligated to pBBR–*lacZ*	This work
P*_wspB_*::*lacZ*	Reporter plasmid with *wspB* promoter ligated to pBBR–*lacZ*	This work
P*_wspC_*::*lacZ*	Reporter plasmid with *wspC* promoter ligated to pBBR–*lacZ*	This work
P*_wspD_*::*lacZ*	Reporter plasmid with *wspD* promoter ligated to pBBR–*lacZ*	This work
P*_wspF_*::*lacZ*	Reporter plasmid with *wspF* promoter ligated to pBBR–*lacZ*	This work
P*_wspR_*::*lacZ*	Reporter plasmid with *wspR* promoter ligated to pBBR–*lacZ*	This work
pBAK-*rpoS*	RpoS expression plasmid in which the *rpoS* gene was under the control of pBAD promoter	This work
pBBR403-*egfp*	Derived from pBBR1MCS5, containing an *egfp* gene controlled by a *tac* promoter	This work

### Plasmid and strain construction

The routine cloning of DNA fragments into plasmid followed a T5 exonuclease-dependent method (44). All PCR cloning steps were verified by commercial sequencing (Tsingke, Wuhan, China). Heat shock transformation or electroporation was used to transfer plasmid to *E. coli* and *P. putida* strains. To construct a promoter-*lacZ* fusion reporter plasmid, we amplified a fragment (about 500 bp) containing the target promoter. We then ligated the fragment into plasmid pBBR-*lacZ*, which harbored a promoterless *lacZ* gene. A fragment containing a mutated *wspA* promoter was generated by overlapping PCR. Briefly, two fragments were generated with primers 1488pros/1488proMa and 1488proMs/1488proa. The 1488proMa and 1488proMs were reverse complementary sequences containing the mutation in which the RpoS motif was changed from CTATACT to TCGGCAC. The two fragments were mixed in a 1:1 ratio to perform overlapping extension. The final PCR product was cloned into pBBR-*lacZ* to yield pBBR-P*_wspA_*_(-10M)_-*lacZ*. The mutation in the *wspA* promoter was confirmed by sequencing. To construct a *rpoS* expression plasmid, we amplified a fragment containing the coding sequence of RpoS. We then ligated the fragment into plasmid pRKara to yield pRKara-*rpoS*, in which the expression of *rpoS* was controlled by an inducible pBAD promoter. Primers used for plasmid and strain construction are listed in Table 2.

**TABLE 2:** Primers used in this work^*a*^

Primer	Sequence (5′→3′)	Purpose
QpcrrpoDs	CCTGATCCAGGAAGGCAACAT	qPCR internal standard
QpcrrpoDa	CAGGTGGCATAGGTCGAGAACT	qPCR internal standard
QpcrwspRqs	GCACGCATCCGTTATCACTC	qPCR primer
QpcrwspRqa	GCCACTCCAGCTCCAGGTAC	qPCR primer
Ct-1488Adw	AGGTGGGCGAGCAACTG	Co-transcription
Ct-1489Bup	CCACGATGGGCGAGAAT	Co-transcription
Ct-1489Bdw	CGGACGAGTTCCAGCC	Co-transcription
Ct-1490Cup	GTTGAGCCAGCCGTTTAT	Co-transcription
Ct-1490Cdw	GTGTACTACTGGCTGGGTTTG	Co-transcription
Ct-1491Dup	GACGGAACAGCAGCATCG	Co-transcription
Ct-1491Ddw	GATGGCTGTCGAGGAAATC	Co-transcription
Ct-1492Eup	CAATACGCATCAGCAGGTC	Co-transcription
Ct-1492E-w	TAGTCCGCAAACGCATCC	Co-transcription
Ct-1493Fup	TCGACACCGTCCATCACC	Co-transcription
Ct-1493Fdw	ATGTGGACCAGGTGTTTGC	Co-transcription
Ct-1494Rup	GCACCATCGCCGAGTTT	Co-transcription
Aq-1488s	CACCATCCAGGACAAAGACG	Absolute qPCR
Aq-1488a	GGCGTTGAGCGACAGCA	Absolute qPCR
Aq-1489s	CGAGATCCAGTCGGCAGTG	Absolute qPCR
Aq-1489a	GCAAGAAGCGGAAGAAACG	Absolute qPCR
Aq-1490s	TAAACGGCTGGCTCAACTGG	Absolute qPCR
Aq-1490a	GCATCGCCTTCGGTATCACT	Absolute qPCR
Aq-1491s	TGCTCAGTGATACCGAAGGC	Absolute qPCR
Aq-1491a	CACGGCAGACAGTAAGTGTTGAT	Absolute qPCR
Aq-1492s	CCGCCTCAATAGCCTGCTT	Absolute qPCR
Aq-1492a	CCTGCCACCTGGGTCAACT	Absolute qPCR
Aq-1493s	TAAAGACCGTGAGGAAGACCG	Absolute qPCR
Aq-1493a	TCTGTAGCAGGCGAATGTGG	Absolute qPCR
Aq-1494s	GCCCACGGTCATTCTTCAA	Absolute qPCR
Aq-1494a	GATGCCGACTTGGTTACGC	Absolute qPCR
Qpcr-1488s	CAGTCAACACGGCAAAGGG	qPCR primer
Qpcr-1488a	AACGCAGCTTGTCGAGGG	qPCR primer
Qpcr-1489s	GCACGAGGTGATCGAAGTGT	qPCR primer
Qpcr-1489a	AGCTCAAGGCGGCAAGG	qPCR primer
Qpcr-1490s	CATCAGCCCAAGTTCGGTAG	qPCR primer
Qpcr-1490a	GGTCAAGCACATTGCCCAC	qPCR primer
Qpcr-1491s	CATTCATTGCCGCACCAG	qPCR primer
Qpcr-1491a	CTCGACAGCCATCACCACC	qPCR primer
Qpcr-1492s	GGAGCTGATCGACGATGGC	qPCR primer
Qpcr-1492a	TGTCACGCAGGCTGAAACC	qPCR primer
Qpcr-1493s	GGCAATCGGGTCTTCGG	qPCR primer
Qpcr-1493a	CGCTGCTGAGCCATTCG	qPCR
EMSA-1488s	ATGGGCATATCGACGAGC	EMSA fragment
EMSA-1488a	TGTCAAGCTACCTGCTGAGCGCAAGGTCCAGTTCTT	EMSA fragment
EMSA-1493a	GGGCATGTCATTGACGATG	EMSA fragment
EMSA-1493s	TGTCAAGCTACCTGCTGAATGATGGCTCGGTGGTCC	EMSA fragment
FAM-tail	TGTCAAGCTACCTGCTGA	Fluorescent label
1488pros	CTGATGCCGGTACCATGGGCATATCGACGAGC	Promoter amplification
1488proMa	GTGCCGACCCAGAGGTCT	Mutated promoter
1488proMs	AGACCTCTGGGTCGGCAC	Mutated promoter
1488proa	TTAGTCATCTGCAGGCGCAAGGTCCAGTTCTT	Promoter amplification
1489pros	CTGATGCCGGTACCAGCCAGCAACATCACCCA	Promoter amplification
1489proa	TTAGTCATCTGCAGTGTTTCCTGTTTGAAGCGCGACACGC	Promoter amplification
1490pros	CTGATGCCGGTACCGGGGCGTTGTACCTGCA	Promoter amplification
1490proa	TTAGTCATCTGCAGTGTTTCCTGTGTCGGGTCTTGCGTGTAC	Promoter amplification
1491pros	CTGATGCCGGTACCCGCCCAAACTGCCAAG	Promoter amplification
1491proa	TTAGTCATCTGCAGTGTTTCCTGTGTTCATCGTTCAGACTCCC	Promoter amplification
1492pros	CTGATGCCGGTACCCGGATGCGGAAAACACC	Promoter amplification
1492proa	TTAGTCATCTGCAGTGTTTCCTGTTGCACGGCAGACAGTAAGT	Promoter amplification
1493pros	CTGATGCCGGTACCATGATGGCTCGGTGGTC	Promoter amplification
1493proa	TTAGTCATCTGCAGTGTTTCCTGTCCGATCAGTTCCACAACAG	Promoter amplification
1494pros	CTGATGCCGGTACCCCGAATGGCTCAGCAG	Promoter amplification
1494proa	TTAGTCATCTGCAGTGTTTCCTGTCGATCACTACCGGCCTG	Promoter amplification

^*a*^ The protective bases and restriction site regions of the primers are indicated by underlining.

### Gene co-transcription assay using reverse transcription PCR

Total RNA from exponentially growing cells was extracted with a total RNA extraction reagent (Vazyme R401-01, China) as recommended by the manufacturer. Contaminated genomic DNA digestion and reverse transcription were performed using a PrimeScript RT reagent kit (Takara RR047A, Japan). Primers used for RT-PCR were designed at the junction between two genes (Table 2). Original RNA without DNA digestion was used as a template in the positive control group, and DNase-treated RNA was used in the negative control group. In the experimental group, cDNA was used as a template. PCR procedure was set as follows: reactions degenerated at 95°C for 30 s, annealed at 55°C for 30 s, extended at 72°C for 30 s, and under 30 cycles. Products were resolved by 1% agarose gel, stained with ethidium bromide, and digitized using a scanner (Tanon 2500, China).

### Compare gene transcription levels using quantitative PCR

Total RNA extraction, contaminated genomic DNA digestion, and reverse transcription were performed as described above. When tetracycline was involved in the qPCR assay, strains were precultured in LB medium without tetracycline for 6 h. Then, tetracycline (final concentration 6 µg/mL) was added to the culture, and strains were incubated for another 3 h before the cells were harvested for RNA extraction. An equal amount of solvent (ethanol) was added to the culture for the control group. Primers used for qPCR are listed in Table 2. AceQ qPCR SYBR Green Master Mix (Vazyme Q121-02, China) was used to quantify the production amount during PCR. The qPCR assay was performed and analyzed using a QuantStudio 3 Real-Time PCR System (Applied Biosystems, USA) to obtain the threshold cycle (Ct) value of each target. PCR procedure was set as follows: reactions degenerated at 95°C for 30 s, annealed at 60°C for 30 s, extended at 72°C for 30 s, and under 40 cycles. For absolute qPCR, each of the seven genes in the *wsp* operon was cloned into plasmid pUC19 before being used as a template in qPCR to generate a standard curve. The standard curves were generated relating the Ct values of the qPCR to the log numbers of target DNA. The absolute number of target genes in cDNA was then calculated according to the standard curve. The copy number of *wspA* was set as 1, and the copy number of the other six target genes was normalized to the copy number of *wspA*. For relative qPCR, the degree of change in the relative quantity of each target gene was calculated using the 2^−ΔΔCt^ method with RpoD used as an internal control for normalization. Three individual replicates were performed with three independent cultures grown on different days (biological repeat) for both absolute and relative qPCR.

### Assays for β-galactosidase activity

Overnight cultures harboring a promoter-*lacZ* fusion reporter were 1:100 diluted into fresh LB medium and incubated at 28°C with 180 rpm shaking. After 10 h of incubation, 2 mL culture was harvested to measure LacZ activity. LacZ activity was measured following a previously described method (45). For assays involved in tetracycline treatment, strains were precultured for 6 h (OD_600_≈0.6) in LB medium without tetracycline. Then, tetracycline (final concentration 6 µg/mL) was added to the culture, and strains were further incubated for 4 h before performing the LacZ activity assay. Measurements were repeated in triplicate with two technical repeats per sample, and data were given in Miller units.

### Biofilm formation analysis with crystal violet staining

The biofilm-forming ability was quantitatively analyzed using crystal violet (CV) staining. Briefly, overnight cultures of *P. putida* were 1:100 diluted into 3 mL fresh LB medium in borosilicate glass tubes and incubated at 28°C with 180 rpm shaking. After 6 h, tetracycline (final concentration 6 µg/mL) or an equal amount of control solvent (ethanol) was added to the culture, and strains continued to grow until the indicated time. After incubation, the cultures were removed, and the OD_600_ of each culture was recorded. Then, biomass attached to the glass surface was stained with crystal violet (0.1%) for 10 min. After staining, excess crystal violet was washed off with tap water three times, and then tubes were left at room temperature for 2 h before taking photographs. The CV that stained the biofilm was dissolved with 95% ethanol by leaving it at room temperature for 20 min. Then, OD_590_ of the ethanol solution containing CV was examined using a spectrophotometer (PerkinElmer, Germany). The final OD value was calculated by dividing the OD_590_ by the OD_600_.

### Biofilm formation in a flow chamber system

The flow chamber system was assembled by modifying the previously described method (46). Overnight cultures of *P. putida* strains harboring plasmid pBBR403-*egfp* were diluted to OD_600_ of 0.01 and injected into the flow chamber channels (six channels per strain). LB medium was continuously imported into the channels at a flow rate of 3 mL/h. After 24 h, tetracycline (final concentration 6 µg/mL) was added to the LB medium of three channels, and strains were incubated for another 24 h. Then, biofilm in the chambers was observed using an FV1000 confocal laser scanning microscope (CLSM) (Olympus, Japan) equipped with a 20×/0.75 objective lens. Thirty-one scanning photos were obtained for each biofilm sample, and the spacing between each scanning was 1 µm. The biofilm structure was rebuilt and displayed using Imaris 9.0.1 software (Bitplane AG, Switzerland). Biofilm biomass was analyzed using COMSTAT2 (47).

### Extraction and quantification of intracellular c-di-GMP

Overnight *P. putida* cultures were 1:100 diluted into 100 mL fresh LB medium in conical flasks and incubated at 28°C with 180 rpm shaking. After 6 h, tetracycline (final concentration 6 µg/mL) or an equal amount of control solvent (ethanol) was added to the culture, and strains continued to grow until the indicated time. After incubation, the cultures were harvested by centrifugation. Intracellular c-di-GMP was extracted and quantitated as previously described (48). Briefly, c-di-GMP was extracted in triplicate from *P. putida* using heat and ethanol precipitation followed by centrifugation. Supernatants were combined, dried using a Speed-Vac, and resuspended in deionized water. The sample was loaded onto a Finnigan Surveyor Plus liquid chromatography system and then onto a Thermo Scientific TSQ Quantum Ultra EMR tandem mass spectroscopy system (LC-MS/MS) (San Jose, USA), which was operated using a selected reaction monitoring mode with the following *m*/*z* transitions: 691.135/135.100 at 51 eV, 691.135/152.000 at 39 eV, and 691.135/248.300 at 25 eV. The daughter ion 152.000 was selected as the quantitative ion. Commercially available c-di-GMP (Biolog Company, Germany) was used as a reference for identifying and quantifying c-di-GMP. c-di-GMP level was normalized to total protein. The experiment was repeated twice with three replicates for each protein.

### Expression and purification of His-tagged RpoS

Overnight culture of *E. coli* BL21 carrying pET28a-*rpoS* was 1:100 diluted into 100 mL LB medium in a conical flask and incubated for 4 h at 37°C with 180 rpm shaking. Then, 0.4 mM IPTG was added to induce the expression of His-tagged RpoS. After 8 h incubation at 16°C with 120 rpm shaking, cells were harvested and resuspended in lysing buffer (10 mM Tris-Cl [pH 7.8], 300 mM KCl, and 10% glycerol). Cells were lysed and filtered through a 0.22-μm-pore-size filter and then loaded to a Ni-NTA column. Wash buffer (10 mM Tris-Cl [pH 7.8], 300 mM KCl, 10% glycerol, and 20 mM imidazole) was added to the column to wash off unspecific binding proteins. Finally, elution buffer (10 mM Tris-Cl [pH 7.8], 300 mM KCl, 10% glycerol, and 250 mM imidazole) was used to wash down the target protein. Protein concentration was determined by BCA assay.

### Western blot assay

*P. putida* strains were incubated in the presence and absence of tetracycline as described above for c-di-GMP extraction and quantification. Then, cells were harvested by centrifugation and lysed with a JNBIO pressure cell-breaking apparatus. The same amount of protein (5 µg) from different samples was resolved by a 12.5% SDS–PAGE and electrotransferred onto the PVDF membrane. WspR proteins in the membrane were tested with an anti-Strep II tag mouse antibody. A horseradish peroxidase-conjugated secondary antibody (goat anti-mouse) was used for the chemiluminescent detection of bound ligands for all blot assays. Detection was carried out using Western ECL reagents. Images were digitized using a Tanon 5200 scanner.

### Electrophoretic mobility shift assay (EMSA)

EMSA was used to test the interaction between RpoS and promoter DNA. Fragments of *wspA*/*wspF* were amplified with PCR primers containing a label labeled with the FAM (6-carboxyfluorescein phosphoramidite) at the 5′' end. The fragment was incubated with RpoS in binding buffer (10 mM Tris [pH 7.8], 50 mM KCl, 10 mM MgCl_2_, and 5% glycerol) at 25°C for 30 min. After incubation, reaction mixtures were loaded onto a 5% acrylamide gel and electrophoresed on ice for 90 min at 100 V in 0.5 × TBE buffer (45 mM Tris-Cl [pH 7.8], 45 mM borate, and 1 mM EDTA). Gels were exposed to a fluorescent imaging system (FLA-5100, FILIFILM) to obtain digital images.

### Statistical analysis

To analyze the significance of differences in biofilm biomass, gene expression, LacZ activity, and c-di-GMP level, Student’s *t*-test was used to compare two data groups, and ANOVA was used to compare three or more data groups. A *P* value less than or equal to 0.05 was considered statistically significant.

## Data Availability

All relevant data supporting the critical findings of this study are available within the article.
